# Topical Therapeutic Options in Corneal Neuropathic Pain

**DOI:** 10.3389/fphar.2021.769909

**Published:** 2022-01-31

**Authors:** Jeremy Nortey, David Smith, Gerami D. Seitzman, John A. Gonzales

**Affiliations:** ^1^ School of Medicine, University of North Carolina, Chapel Hill, NC, United Statesa; ^2^ Francis I. Proctor Foundation, University of California, San Francisco, San Francisco, CA, United States; ^3^ A&O Compounding Pharmacy, Vallejo, CA, United States; ^4^ Department of Ophthalmology, University of California, San Francisco, San Francisco, CA, United States

**Keywords:** corneal neuropathic pain, neuropathic ocular pain, low-dose naltrexone, lacosamide, sub-basal corneal nerve plexus, serum tears

## Abstract

**Purpose of Review:** Corneal neuropathic pain can be difficult to treat, particularly due to its lack of response to standard dry eye therapies. We describe a variety of topical therapeutic options that are available to treat corneal neuropathic pain with a significant or primary peripheral component. We also describe possible mechanisms of action for such topical therapies.

**Recent Findings:** Topical corticosteroids and blood-derived tear preparations can be helpful. Newer therapies, including topical lacosamide and low-dose naltrexone are emerging therapeutic options that may also be considered.

**Summary:** Corneal neuropathic pain with a significant peripheral component may be managed with a variety of topical therapeutic options.

## Introduction

Corneal neuropathic pain has perplexed ophthalmologists particularly because of its symptomatic masquerade as dry eye disease. Indeed, some patients with corneal neuropathic pain may have dry eye findings that are compatible with evaporative and/or aqueous deficient dry eye disease. However, these patients fail to respond to dry eye treatments (Galor et al., 2015a; Galor et al., 2016). Some patients may even be suspected of malingering or of having somatic symptom disorders. Patients often have seen a variety of ophthalmologists for second, third, or more opinions. While corneal neuropathic pain can certainly exist in the setting of aqueous sufficient and deficient dry eye, the common clinical situation encountered is a relatively unremarkable ocular surface examination in a person complaining of significant ocular discomfort (the so-called “pain without stain” patient) (Rosenthal et al., 2009). Increasing awareness of the existence of corneal neuropathic pain, a distinct clinical entity from aqueous sufficient and aqueous deficient dry eye disease, can benefit patients by advancing them on a more directed course to more specifically addresses their discomfort (Craig et al., 2017). Management of neuropathic pain, however, continues to be a challenge, in part, due to a lack of comparative clinical trials identifying the most effective treatment strategies. Often, determining whether there is a primarily peripheral or a primarily central component of neuropathic pain (or perhaps, mixed) can identify routes of therapy that may be most efficiacious (Rosenthal et al., 2009; Rosenthal and Borsook, 2012; White et al., 2014; Dieckmann et al., 2017; Ozen et al., 2017). In patients exhibiting a significant peripheral component of corneal neuropathic pain or discomfort, topical therapeutic approaches may be of benefit (Asbell, 2006; Mondy et al., 2015; Chen et al., 2019; Siedlecki et al., 2020). This paper will use both the terms “pain” and “discomfort” to describe the subjective complaints of patients suffering from corneal neuropathic pain. Some patients point out that they do not experience “pain” per se, but rather a sensation that is often difficult to define but is, nevertheless, uncomfortable.

The nerves within the cornea stem from branches of the nasociliary nerve which itself is a branch of the ophthalmic division of the trigeminal nerve also known as the first division of the fifth cranial nerve. Nasociliary nerve branches enter the peripheral cornea radially before traveling anteriorly to penetrate Bowman’s layer and forming the sub-basal nerve plexus. Ocular surface pain and corneal neuropathic pain is initiated by these peripheral nerves and their free endings located within the cornea (Mehra et al., 2020). Other pain nerve fibers, called nociceptor fibers, variably respond to different stimuli. Mechanoreceptors are responsible for the sharp pain that is felt upon mechanical stimulation such as an object coming in contact with the cornea. Polymodal receptors respond to mechanical, thermal, pH, and chemical changes within the environment, and are associated with burning and stinging pains. Cold thermoreceptors respond to changes in temperature and tear osmolarity, and can be associated with ocular surface discomfort that may be generated with evaporative tear (Rosenthal and Borsook, 2012; Cho et al., 2019; Mehra et al., 2020). Because of its peripheral location in the cornea, the sub-basal nerve plexus is the peripheral-most component to efferent and afferent corneal neuropathic pain. However, there is also a significant central component to corneal neuropathic pain, which involves first- and second-order nerve input to the thalamus and, ultimately, the cortex with additional modulation from the thalamus and amygdala (Rosenthal and Borsook, 2012; Mehra et al., 2020).

Our group at the Proctor Foundation at the University of California San Francisco serves as a major tertiary center for the evaluation and management of patients with corneal neuropathic pain. Herein, we discuss the topical (eye drop) therapeutic options that are suggested for patients with a component of peripheral corneal neuropathic pain. Our experience has been that patients with peripheral corneal neuropathic pain are very motivated to understand what eye drop therapies may be of potential benefit to them. While there are a variety of eye drops that can be used in corneal neuropathic pain, no randomized controlled trials comparing one class of eye drop to another in corneal neuropathic pain currently exists. Commercially available and compounding pharmacy-only available drops are reviewed. To ensure a comprehensive review, we searched for topical (eye drop) therapeutics in PubMed using search terms, “neuropathic pain”, “ocular pain”, and “eye pain” and evaluated the results if the abstracts described topical therapeutics. A total of 204 records were screened and 182 records were excluded. Of the 22 reports assessed for eligibility, additional records were excluded due to focusing on post-operative treatments (4), focusing on systemic treatments (2), and a lack of relation to neuropathiic ocular pain (6). Ten studies were included in this review. Our group’s experience in topical therapy was also included (Table 1).

**TABLE 1:** Topical therapeutic options available as eye drops for peripheral corneal neuropathic pain.

Topical therapies	Mechanism of action
Corticosteroids	Anti-inflammatory properties can decrease the density of dendritic cells
Blood-derived tear preparations	Autologous or allogenic tear promote corneal epithelial cell health and are associated with fewer features of neuropathy of sub-basal nerve plexus
Lacosamide	Amino acid molecule that decreases hyperexcitability of corneal cold-sensitive nerve terminals
Low-dose naltrexone	Opioid antagonist known for its effects on bodily neuropathic pain
Enkephalin modulators	A relatively new therapy that acts as a neuropeptide inhibitor with effects on modulating pain

## Assessment of Corneal Neuropathic Pain

The assessment of corneal neuropathic pain involves 1) identifying the presence of subjective ocular pain and, if possible, 2) identifying features that align with a neuropathic phenotype. Questionnaires and *in vivo* confocal microscopy can be helpful in distinguishing patients with corneal neuropathic pain from those with non-neuropathic ocular discomfort that can be associated with dry eye disease. Patients may have features of both an aqueous sufficient or aqueous deficient dry eye disease in addition to frank corneal neuropathic pain.

Questionnaires that are specific for ocular pain can be easily self-administered in the clinical setting or administered by staff and include the Ocular Pain Assessment Survey (OPAS) and the Neuropathic Pain Symptom Inventory modified for ocular pain (NPSI-Eye) (Qazi et al., 2016; Farhangi et al., 2019).

Prior to 2016, there was no standardized and validated way of assessing ocular pain intensity and aggravating factors. The OPAS was a major step forward in identifying, anatomically locating and quantifying the intensity ocular pain. Comprised of 27 questions, spanning a gold-standard visual analog system of quantifying pain intensity (Wong-Baker FACES pain rating scale), aggravating factors, and quality of life features, the OPAS is not only comprehensive but useful in monitoring response to therapy (Qazi et al., 2016). The NPSI attempts to identify neuropathic-specific ocular pain symptoms, including burning sensation (Bouhassira et al., 2004; Farhangi et al., 2019). The identification of specific neuropathic pain features is important because such patients may be less apt to respond to artificial tears, which may be helpful in patients with non-neuropathic ocular discomfort (Galor et al., 2015b).

Following questionnaires, a critical component of the clinical examination is determining a patient’s subjective response to topical anesthetic. Complete improvement in discomfort or pain is in keeping with a primarily peripheral corneal neuropathic pain process. If some component of discomfort persists, this may suggest that there is a component of the pain that is central while a complete lack of response to topical anesthetic suggests that the discomfort is primarily central. For those corneal pain processes that have at least some component of a peripheral phenotype, topical pain modulatory therapies may be of benefit. Herein, we discuss a variety of topical therapeutic options that are available to clinicians for managing peripheral corneal neuropathic pain.

### Topical Corticosteroids

In both dry eye disease and neuropathic ocular pain, inflammatory cells may mediate a component of the discomfort experienced by patients. Antigen-presenting cells, including dendritic cells, can be found within the corneal epithelium and stroma, and are important players in the complex immune functions and responses exhibited by the cornea in both processes (Hamrah et al., 2003). Compared to control patients, *in vivo* corneal confocal microscopy demonstrates a higher density of dendritic cells in patients with dry eye and corneal neuropathic pain (Kheirkhah et al., 2015; Shetty et al., 2016a; Nicolle et al., 2018). Moreover, in some cases, particularly in aqueous deficient dry eye disease, dendritic cells may be larger and be composed of more individual dendrites than in aqueous sufficient dry eye disease (Kheirkhah et al., 2015). Nicolle et al. (2018) To counterbalance this, endogenous peptides, known as enkephalins, serve to mitigate pain. Moreover, notable effects on *in vivo* confocal microscopy show a decrease in density of dendritic cells after treatment (Villani et al., 2015). Dendritic cells may express inflammatory cytokines and enzymes that degrade neuropeptides, thereby augmenting corneal nociceptors which are involved in initiating the perception of pain (Shetty et al., 2016a; Shetty et al., 2016b; Khamar et al., 2019). Indeed, it is hypothesized that dendritic cell signaling may be involved in mediating and enhancing nociceptive properties in a variety of dry eye, corneal neuropathic pain, and systemic neuropathy diseases and syndromes (Klitsch et al., 2020). Animal models of dry eye disease have demonstrated that dendritic cells can secrete a variety of proteins that activate downstream chemokines and cytokines that can modulate inflammation on the ocular surface (Gandhi et al., 2013; Zhang et al., 2014). Topical corticosteroids have well known anti-inflammatory effects. Thus, decreasing the density of dendritic cells with topical corticosteroids is one approach that may help in cases of corneal neuropathic pain where confocal microscopy identification of sub-basal dendritic cells is a predominant feature (Villani et al., 2015).

### Blood-Derived Tear Preparations

Blood-derived tear preparations contain various growth factors, vitamins, and cytokines and could be considered more similar to natural tears than commercially available over-the-counter artificial tear products. Such tear preparations can be derived from a patient’s own serum as autologous serum tears, from platelet rich plasma, or from allogeneic sources, such as donor cord blood (Tsubota et al., 1999a; Wu et al., 2021). Serum has been shown to promote the differentiation of corneal and conjunctival epithelial cells to express mucins (Gipson et al., 2003). This allows for the migration of corneal epithelial cells in a dose-dependent fashion and may, in part promote corneal epithelial health by stimulating expression of other growth factors and receptors (Phan et al., 1987; Tsubota et al., 1999a; Tsubota et al., 1999b; Geerling et al., 2001). A healthy corneal epithelium helps protect the sub-basal nerve plexus.

There has been some debate about the efficacy of autologous serum tears for dry eye disease. An extensive review by the Cochrane Database Group to identify randomized controlled trials evaluating the efficacy of autologous serum tears compared to artificial tears did not find significant evidence of a long-term durable benefit of autologous serum tears (Pan et al., 2017; Aggarwal et al., 2019). However, some studies have found in pain associated with dry eye, cord blood is superior to placebo (Campos et al., 2020). In non-randomized, observational series, platelet rich plasma drops helped to alleviate pain in corneal surgery-related corneal trauma (Alio et al., 2018).

Neuropathic ocular pain, which is distinctly different from aqueous deficient or sufficient dry eye disease, may benefit from blood-derived tear preparations that studies of general dry eye disease have not been powered to detect. Indeed, in patients with dry eye disease, serum tears have been shown to decrease basal epithelial cell density on confocal microscopy (Mahelkova et al., 2017). While some studies using serum tears in dry eye disease did not identify significant changes in the number of Langerhans cells or activated keratocytes or in the features of the sub-basal nerve plexus, other studies found that corneal nerve morphology improved with fewer neuropathic features using blood-derived tear products (Giannaccare et al., 2017; Mahelkova et al., 2017). The average concentration of serum growth factors vary according to patients who suffer from numerous systemic disease as comparted to healthy subjects with only ocular surface disease. This may be one reason explaining some therapeutic variability of serum tears in different patient populations (Ripa et al., 2020; Siedlecki et al., 2020).

In corneal neuropathic pain, serum tears have been described to be helpful in patients experiencing discomfort to light (photoallodynia) (Aggarwal et al., 2015). Autologous serum has been shown to decrease findings of sub-basal corneal nerve beading and neuromas (Aggarwal et al., 2015). In addition, serum tears have been associated with an improvement in other nerve metrics (as assessed by semi-automated quantification by ImageJ analysis) including total nerve length, nerve number, decrease in nerve reflectivity, and decrease in nerve tortuosity (Aggarwal et al., 2019). Such improvements in nerve metrics have also been correlated with an improvement in ocular pain by patients, suggesting that the resolution of neuropathic features is associated with an improvement in corneal nerve function (Aggarwal et al., 2019). Fresh frozen plasma and platelet-enriched plasma have similar purported benefits as serum tears and are worthy of future inverstigation with regard to ocular neuropathic discomfort/pain (Wang et al., 2021).

### Topical Lacosamide

Lacosamide (C_13_H_18_N_2_O_3_, molecular weight 250.29 g/mol, pubchem.ncibi.hlm.nih.gov accessed August 1, 2021) is an amino acid molecule that was originally developed as an antiepileptic medication. Its main mechanism of action is to selectively enhance the slow inactivation (as opposed to the fast inactivation) of voltage-gated sodium channels (Errington et al., 2008; Niespodziany et al., 2013; Rogawski et al., 2015). Lacosamide also binds with the collapsin-response mediator protein-2 (CRMP-2), involved in modulating neurite outgrowth, which is important in establishing new neuronal projections in developing neurons as well as in regenerating nerves (Wilson and Khanna, 2015; Wang et al., 2018).

While corneal pain is sensed by mechano-nociceptors and polymodal nociceptors, cold thermoreceptors also play a role in the perception of ocular pain and discomfort (Belmonte and Gallar, 2011). Modulation of cold thermoreceptors, then, has been a potential therapeutic target. Topical lacosamide, in an *ex vivo* model, has been shown to decrease the hyperexcitability of corneal cold-sensitive nerve terminals (Kovács et al., 2016). In an aqueous deficient animal model (in which the lacrimal glands had been extirpated), the hyperexcitability as measured by the nerve terminal impulses in corneal cold-sensitive nerve terminals was decreased (Kovács et al., 2016).

Lacosamide 1% is produced from preservative-free Vimpat (UCB Inc., Smyrna, Georgia) 10 mg/ml 20 ml vial. It is important to note that Vimpat is a Schedule 5 drug and all state and federal laws and regulations regarding controlled substances must be followed when prescribing and dispensing this drug. The compounding of lacosamide 10 mg/ml is an aseptic transfer from the injection vial to the droptainers or dispensing devices. When assigning a beyond use date, pharmacies must follow corresponding state and United States Pharamcopeia (USP) General Chapter 797 regulations. According to USP General Chapter 797, a beyond use date of 14 days refrigerated may be assigned. The package insert for Vimpat states that it is not to be frozen. The product Vimpat 20 mg/20 ml is available through drug wholesalers only as packs of 10. It is important to find a pharmacy that is willing to make the initial investment in order to stock the medication for compounding use.

### Topical Low-Dose Naltrexone

Naltrexone (C_20_H_23_NO_4_, molecular weight 341.4 kg/mol, pubchem.ncibi.hlm.nih.gov accessed August 1, 2021) is an opioid antagonist that was originally developed as a therapeutic for opioid and alcohol addiction, typically at oral doses from 50 to 100 mg daily. Low-dose naltrexone (typically in doses from 1 to 5 mg daily) has been used for treating bodily neuropathic pain (Younger and Mackey, 2009; Metyas et al., 2018). Ultra-low-dose naltrexone (doses below 1 mg daily) may also be used (Toljan and Vrooman, 2018).

Naltrexone is noted to have effects on opioid receptors (commonly described with mu-opioid receptors as well as others) and non-opioid receptors. A non-opioid receptor that seems to be associated with the functionality of naltrexone as it pertains to pain modulation is the Toll-like receptor, which is found on macrophages and microglia. Microglia are involved in pain through the binding of a protein, high mobility group box 1, HMGB1) which binds to Toll-like receptors 2 and 4 (Watkins et al., 2007). Toll-like receptor expression has been noted to be upregulated in neuronal injury (Owens et al., 2005). Microglia may then increase the expression of tumor necrosis factor-alpha (TNF-alpha) and other inflammatory mediators *via* Toll-like receptor as well as NF-ΚB signaling, indicating a close relationship between inflammation and pain (Nadeau and Rivest, 2000; Thibeault et al., 2001). Ultimately, glial activation is thought to enhance neuroexcitability, which may be associated with the increased perception of pain (Watkins et al., 2007). Naltrexone has been shown to inhibit the IL-6 and TNF-alpha that is produced after Toll-like receptors have interacted with their cognate ligands, which can thereby mitigate pain in animal models (Grace et al., 2015; Cant et al., 2017).

Models of ocular surface injury (penetrating trauma or alkali injury) have been associated with retinal damage and inflammation within the brain by virtue of microglial and macrophage activation (Ferrari et al., 2014; Paschalis et al., 2018). This has suggested that modulation of ocular surface neuropathy can be beneficial in a wide array of conditions. Indeed, oral low-dose naltrexone has been described to be beneficial to patients with a central component of neuropathic ocular pain. Patients treated with oral low-dose naltrexone as monotherapy or as part of a multimodal therapeutic approach was assocated with a decrease in their mean visual analog pain score as well as decrease in their mean quality of life score as assessed by the OPAS (Dieckmann et al., 2021). Topical low-dose naltrexone has been used in a diabetic murine model demonstrating improvements in corneal nerve sensitivity to a filament (von Frey) similar to that used to test human corneal sensation (Cochet-Bonnet enesthesiometer) (Zagon et al., 2014).

Interest in naltrexone’s potential to ameliorate corneal neuropathic problems, such as ulcerations and delayed re-epithelialization, have led to interest in producing contact lenses that would allow for the ability to deliver a more constant level of low-dose naltrexone to the cornea’s sub-basal nerve plexus (Alvarez-Rivera et al., 2019).

Presently Naltrexone, as an eye drop, requires specialty compounding. Naltrexone eye drops are reconstituted from active pharmaceutical ingredient powder because a commercial product does not currently exist. This preparation is considered high-risk compounding because pharmacies start the process with non-sterile powder and sterilize the solution *via* membrane filtration as the final step. Naltrexone HCl USP powder is dissolved in sodium chloride 0.9% sterile injection and preserved with benzalkonium chloride solution (though a preservative-free formulation can be prepared as well). The pH may range from 4.5 to 6.2. The solution is drawn up in a luer lock syringe and sterile filtered into droptainers or dispensing devices. For this type of preparation, USP General Chapter 797 allows a Beyond Use Date of 3 days refrigerated or 45 days frozen. Strengths of naltrexone ophthalmic solution range from 0.001 to 0.2%. The compounding of high-risk preparations is performed in an International Organization for Standardization (ISO) 7 ante room and the final filtering and packaging is done in an ISO 5 laminar flow hood.

### Topical Enkephalin Modulators: Future Targets

Because the neuropeptides known as enkephalins can modulate pain, there has been interest in utilizing this pathway as a potential pain therapeutic. Enkephalins are associated with an analgesic effect, but their action is relatively short-lived due to the presence of enzymes that rapidly degrade these neuropeptides. Endogenous inhibitors of enkephalin enzymes do exist and are present in foreign body models of ocular pain (Ozdogan et al., 2020; Lasagni Vitar et al., 2021). Thus, pharmacologic inhibition of such enzymes may be a therapeutic target. In one study, the topical administration of PL265, an inhibitor of enkephalinase, in a murine model of corneal pain reduced corneal mechanical and chemical hypersensitivity (Reaux-Le Goazigo et al., 2019). Targeting corneal mu-opioid receptors with agonists may be another therapeutic target as suggested in murine models (Joubert et al., 2020).

## Discussion

There has been substantial progress in the development of tools that can identify corneal neuropathic pain (Qazi et al., 2016; Farhangi et al., 2019). The identification of ocular pain and neuropathic pain features with the OPAS and NPSI questionnaires is an important step forward in better distinguishing patients that are less likely to respond to lubrication like their typical dry eye counterparts. However, significant challenges remain, particularly regarding treatment of corneal neuropathic pain. *In vivo* confocal microscopy can be helpful in identifying dendritic or other inflammatory cells, which may suggest a role for a brief course of topical corticosteroids. Autologous blood-derived products are a reasonable first-line approach given their association with improvement in comfort and features of neuropathy on *in vivo* confocal. If there is a significant peripheral component, consideration can be made for other topical therapies such as lacosamide or low-dose naltrexone drops compounded through a specialty pharmacy. While longitudinal comparative studies demonstrating the most effective topical treatment strategies have yet to be performed, it is reassuring that a variety of topical therapeutic options exist for patients with a peripheral component of corneal neuropathic pain and discomfort. A concerted effort on the part of both ophthalmologists and patients offer the possibility of future randomized controlled trials that can provide high-quality evidence for managing corneal neuropathic pain.
